# Comparison of Factors Associated with Fear of Falling between Older Adults with and without a Fall History

**DOI:** 10.3390/ijerph15050982

**Published:** 2018-05-14

**Authors:** Seonhye Lee, Eunmi Oh, Gwi-Ryung Son Hong

**Affiliations:** 1 Department of Nursing, Gyeongnam National University of Science and Technology, #33 Dongjinro, Jinju, Gyeongnam 52725, Korea; shlee@gntech.ac.kr; 2 School of Nursing, Hanyang University, #222 Wangsimliro, Sungdong-gu, Seoul 04763, Korea; grson@hanyang.ac.kr

**Keywords:** fear of falling, older adults, environmental factors, ecological model

## Abstract

*Background*: Although fear of falling (FOF) has been studied since FOF has negative consequences for the elderly, there is limited information about the risk factors of FOF, including the environment. The purpose of this study was to describe individual and environmental factors of FOF between those with and without a fall history from an ecological aspect and to examine whether individual and environmental factors differently affect the FOF according to the state of fall history in community-dwelling older adults in Korea. *Methods*: Data from the 2014 Survey of Living Conditions and Welfare Needs of Korean Older Adults were used. Participants were 7730 older adults. Hierarchical logistic regression analysis was conducted to examine the predictors of FOF. *Results*: According to the ecological model, female and discomfort with the neighborhood environment were significantly associated with greater odds of reporting FOF in both older adults with fall history and those without. A significant interaction was not observed between any variable of FOF in participants with and without a fall history. *Conclusions*: An ecological model including individual and environmental factors should be considered when conducting research and designing programs and decision policies related to FOF for older adults with and without a history of falling.

## 1. Introduction

Falls are a common problem in community-dwelling older adults [1] and are a leading cause of adverse health outcomes such as fall-related injuries [1,2], hospitalization [3], and death [3,4]. The increasing medical expenses and burden of falls constitute a global problem, and fall incidence and total cost of fall increase with age [5,6]. In Finland and Australia, the average direct cost to the health care system per one fall for people 65 years or older was US$ 3611 and US$ 1049, respectively; in the UK, the average indirect cost of falls per year, including loss of productivity of family caregivers, was about US$ 40,000 [5]. Hence, establishing a multi-faceted and comprehensive fall prevention strategy in the elderly is a very important global health issue [5].

Fear of falling (FOF) is a risk factor for falls [2] and is associated with negative physical and psychosocial health outcomes, including depression [7] and activity restriction [8,9,10,11]. Based on a seven-year follow-up longitudinal study [4], FOF increased the rate of death among community-dwelling older adults. Based on recent longitudinal studies, FOF is a prevalent phenomenon among community-dwelling older adults where the incidence of FOF was 56.7% in one study [12] and 75.6% in another [13]. Therefore, research focusing on health care in older adults has recently expanded to identify the determinants of FOF.

Recently, a number of studies have investigated the individual-level factors and predictors of FOF among older adults [14]. Individual-level predictors consist of multidimensional constructs of FOF such as general characteristics as well as physical, psychosocial, and health-related variables [13,15]. Being female, having poor physical function, using a walking aid, having a fall history, having poor self-rated health, having depression or anxiety, using multiple drugs, and being on psychotropic drugs were found to be associated with FOF according to a systematic review [16]. Among individual factors, identification of modifiable factors such as fall history of FOF is important for effective health promotion by preventing negative health outcomes in old adults. In a systematic review of FOF in older adults [17], the important factors were reported as fall history (at least one), female, and older. Of these, the only factor that can be modified through intervention is fall history. The history of falls was consistently reported as a risk factor of FOF in previous studies [13,15,18], and the risk of FOF was 6.41 times higher in community-dwelling older adults with a history of falling than in those with no such history [15]. Thus, a better understanding of the influences of fall history status on FOF is crucial in planning intervention to prevent FOF among older adults.

However, other evidence suggests that environmental factors should be equally considered [14]. Environmental risk factors in the fall risk model include homes and neighborhood environments such as unsafe sidewalks and inadequate lighting [19]. Recent studies have demonstrated that an uncomfortable neighborhood environment for older adults reduces physical activity, increases the risk of falls, and ultimately lowers quality of life [20,21,22]. Current fall prevention guidelines of the American Geriatrics Society recommend FOF screening for prevention of falls [23]. Nevertheless, only a few studies have reported environmental factors related to FOF: lack of social interaction with family and friends [12], residential area (rural or smaller city) [14], and difficulty using public transportation [24]. A qualitative study mentioned that older adults were less like to walk around if they felt unsafe, for example, on wet subways floors and sloped walkways [25]. Previous research included limited elements of environmental factors on FOF [12,14,26], though did not take into account some individual factors such as depression [14].

There were several studies using national data to identify factors influencing FOF; however, no study has examined the differences in predictors of FOF between those with and without fall history and included environmental factors in their model. Strategies to prevent FOF should be comprehensive and multifaceted [13]. Especially, environmental factors are difficult to be changed and cannot typically be solved by individuals. Therefore, it is necessary to support policies for the establishment of safe environments and to explore individual factors. Korea is one of several countries that is facing the problem of rapid growth in the aging population; fall accidents have not only increased incrementally [27], but FOF was 78.7% in a 2014 national survey on the living status and welfare needs of the elderly [28]. Therefore, the purpose of this study was to describe individual and environmental factors of FOF between those with and without a fall history using ecological aspects and to examine whether individual and environmental factors differently affect FOF according to fall history in community-dwelling older adults in Korea.

## 2. Materials and Methods

### 2.1. Description of Data Sources

This study is a secondary analysis of data from the 2014 National Survey of Living Conditions and Welfare Needs of Korean Older Adults. The analysis was performed to understanding of FOF in older adults to inform evidence-based policy. During the period from June 11th to September 4th, 2014, trained investigators visited and interviewed older adults over 65 years old. These data have been described in detail elsewhere [28].

### 2.2. Design and Sample

**Figure 1:**
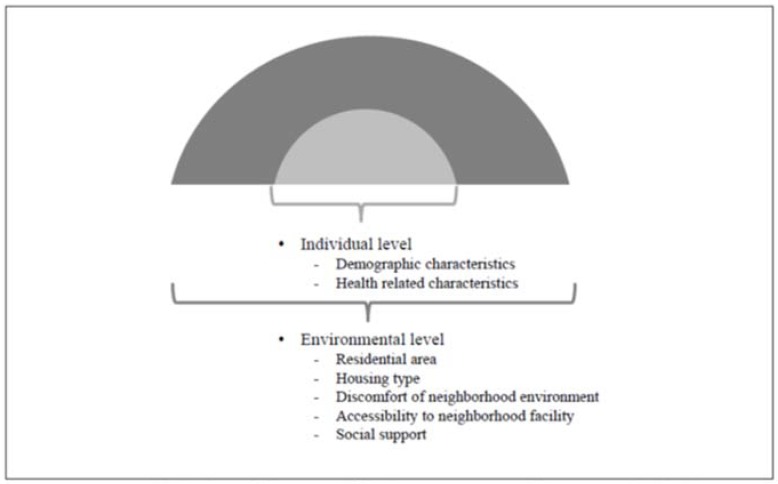
Research design of factors affecting FOF according to ecological approach.

This study was a descriptive correlational study investigating the predicting factors of FOF in older adults using the ecological model (Figure 1). In 2014, a cross-sectional survey was conducted in residential areas including 16 cities and provinces. To optimize the reliability of our analysis, those who did not respond to FOF (*n* = 174) and those with impaired cognitive function (*n* = 2547) were excluded. Cognitive impairment was evaluated using the Korean version of the mini-mental state examination for dementia screening (MMSE-DS) and was noted if the score was less than −1.5 SD based on the guidelines considering sex, age, and education [29]. A total of 7730 older adults were selected from the original data of 10,451 participants who responded to the survey in 2014.

### 2.3. Ethical Considerations

The 2014 Survey of Living Conditions and Welfare Needs of Korean Older Persons was conducted under the approval of the National Statistical Office (Approval No. 11771). The original data were provided without personal identification information. The Public Institutional Bioethics Committee designated under the Ministry of Health and Welfare approved waiver of consent for this study (Institutional Review Board No. 2017-0485-001).

### 2.4. Measurements

#### 2.4.1. Fear of Falling

FOF was investigated using the question, ‘Are you usually afraid of falling’? Participants were asked to choose one in a three-point scale: not at all, some, or a lot, with a higher score indicating a greater FOF. ‘Not at all’ was taken as ‘no FOF’, while some and a lot were taken as ‘have FOF’ in data analysis [12,13,30].

#### 2.4.2. Individual Level Characteristics

The individual level characteristics included demographic and health-related characteristics. First, demographic characteristics included age (years), sex, marital status (living with spouse or living without spouse), education level (0–6 years or over 7 years), public assistance (yes or no), and current driving status (yes or no). Second, the health-related characteristics of the participants were subjective health status, limitation of activities of daily living (ADL), limitation of instrumental activities of daily living (IADL), troubles with vision and hearing, hypertension, diabetes mellitus, arthritis, and depression. Subjective health status was assessed using the question, ‘What do you think is your normal health condition’? Participants were asked to choose among very healthy, healthy, average, bad, or very bad. ‘Very healthy’ and ‘healthy’ were re-coded as ‘healthy’, and the rest was categorized ‘not healthy’ in this analysis.

*Limitation of ADL* was determined using the Korean ADL scale (K-ADLs) [31] consisting of seven questions on a three-point scale. A higher score indicates no ADL limitation; total independence was re-coded as ‘independent’, and partial and complete dependence were categorized as ‘dependent’. *Limitation of IADL* was determined using the Korean IADL scale (K-IADLs) [31] consisting of 10 questions on a three-point scale. A higher score indicates no IADL limitation; total independence was re-coded as ‘no limitation (independent)’, and partial dependence and complete dependence were categorized as ‘limitation (dependent)’. *Trouble with vision and hearing* was measured using the question, ‘Do you have any difficulties in your daily life due to problems with your vision or hearing’? Participants were asked to choose among no difficulties, difficulties, and many difficulties. Difficulties and many difficulties were categorized into ‘trouble’ and no difficulties were categorized into ‘no trouble’. *Doctor’s diagnosis for hypertension, diabetes mellitus, and arthritis* [27] were categorized into ‘diagnosed’ or ‘not diagnosed’ depending on whether a doctor had diagnosed them with hypertension, diabetes mellitus, and/or arthritis, respectively. *Depression* was assessed using the Short Form of the Geriatric Depression Scale-Korea (SGDS-K) [32], which consists of 15 questions related to depressed mood. Each item was evaluated with 1 point for ‘depressed’ and 0 for ‘not depressed’, and the higher the score, the greater the severity of depression.

#### 2.4.3. Environmental Level Characteristics

The environmental level characteristics were investigated using residential area, housing type, discomfort level in the neighborhood environment, accessibility to facilities in their neighborhood, and social support.

*Residential area* was distinguished by the Korean administrative divisions of urban and rural, and the *housing type* was classified into either apartments or houses.

*Discomfort in neighborhood environment* was evaluated using the overall discomfort perceived by the subject at home, when they go out, and in the community. The answers to these occasions were given as ‘yes, discomfort’ (1 point) or ‘no, no discomfort’ (0 point), so the higher total scores from the three answers indicate a greater level of discomfort in the neighborhood environment. First, discomfort at home [8,20] was determined by identifying uncomfortable living areas such as the entrance/hallway, stairs, bathroom, bedroom, living room, door thresholds, kitchen/dining room, others, or no uncomfortable spaces at the home. Second, the discomfort level when going outside [8,10,20,33] was determined by selecting from the following: getting on and off buses (subways), walking up and down stairs/slopes, lack of transportation, too rough roads to travel, transit amenities without consideration for older adults, dangers from too much traffic, or no discomfort when outside. Third, discomfort level within community [20,33] was determined by selecting among markets/supermarkets, difficulties of access to facilities such as banks, public transportation, green areas/parks, medical facilities, social welfare facilities (including leisure/cultural facilities), others, or no discomfort within the community.

*Accessibility to neighborhood facility* was evaluated by the amount of time it took the participant, on average, to access facilities such as (1) markets/supermarkets; (2) hospitals/clinics/health centers; (3) public offices; (4) senior welfare service centers; (5) other welfare service centers (comprehensive, disabled person)/woman service center; and (6) bus/subway stations. An access time of less than 10 min by walking was categorized as ‘proper’, whereas more than 10 min was taken as ‘not proper’. The answers to those six accessibility questions were given either ‘yes’ (1 point) or ‘no’ (0 point), so a higher score indicated more proper access time to the facilities. In a study on the neighborhood environment of older adults [34], neighborhood was mainly used as an ‘area near the home’ or ‘area within a 10-min walk’. Based on this information, ‘10 min walking’ was evaluated at an appropriate time for accessibility to access neighborhood facilities for this study.

*Social support* [12,20,33] was determined using inquiry on existence of close relatives, close friends/neighbors, and having visitations by people other than cohabitants within the past month. Respondents answered ‘yes’ (1 point) or ‘no’ (0 point). The scores from those three categories were summed to yield a total score of social support, where a higher score indicated greater social support.

#### 2.4.4. Fall History

*Fall history* was evaluated using a single question: ‘Have you experienced a fall (stumble, slip, or fall down) in the past year?’ The respondents could answer ‘experienced a fall’, or ‘did not experience a fall [28]’.

### 2.5. Data Analysis

Data were analyzed using the IBM SPSS statistical software, version 22 (IBM, Armonk, NY, USA). Differences in the FOF by individual and environmental characteristics were identified using the chi-square test or *t*-test. Hierarchical logistic regression analyses were conducted to identify the predictive factors of FOF. Bivariate logistic regression analyses were used to verify the association between each variable and those with and without fall history. The significant variables in the bivariate logistic regression were entered into the hierarchical logistic regression model. In Model I, interaction terms with individual variables and fall history were entered; in Model II, the interaction terms with environmental variables and fall history were entered. Odds ratios (ORs) and corresponding 95% confidence intervals (CIs) were calculated for logistic regression. The level of statistical significance was set at less than 0.05.

## 3. Results

### 3.1. Differences in FOF According to Individual Characteristics of Participants with and without a Fall History

Differences in FOF according to individual characteristics of the participants with and without a fall history are shown in Table 1. In terms of socio-demographic and health-related characteristics, all factors were statistically significant in the two groups (all *p* < 0.01). The prevalence rate of FOF was 96.7% and 75.1% of people with and without a fall history, respectively (not listed in the table).

### 3.2. Differences in FOF According to Environmental Characteristics of the Participants with and without a Fall History

Table 2 shows differences in FOF according to environmental characteristics of the participants with and without a history of falling. There were statistically significant differences in discomfort with the neighborhood environment and social support (all *p* < 0.001), but associations with residential area, house type, and accessibility to neighborhood facilities were not significant.

### 3.3. Associations between Fall History and Individual and Environment Characteristics

Individual and environmental variables associated with a history of fall are shown in Table 3. In both with and without fall history groups, female and discomfort with the neighborhood environment were associated with higher FOF than male and no discomfort, respectively. In the fall history group, only the two factors of female (OR = 5.100, 95% CI = 2.488–10.456) and high discomfort with neighborhood environment (OR = 1.387, 95% CI = 1.026–1.875) were found to be significant for FOF. In those without fall history, nine individual factors comprising 75 years and older (OR = 2.047, 95% CI = 1.738–2.412), female (OR = 3.337, 95% CI = 2.810-3.962), lower level of education (0–6 years) (OR = 1.221, 95% CI = 1.045–1.426), not currently driving (OR = 1.490, 95% CI = 1.250–1.775), poor self-rated health (OR=1.651, 95% CI = 1.418–1.921), trouble with vision (OR = 1.409, 95% CI = 1.206–1.646), diabetes mellitus (OR = 1.295, 95% CI = 1.074–1.562), arthritis (OR = 1.490, 95% CI = 1.240–1.790), and high depression (OR = 1.094, 95% CI = 1.070–1.118) were significant for FOF. Three environmental factors of discomfort with the neighborhood environment (OR = 1.305, 95% CI = 1.211–1.460), high access to neighborhood facilities (OR = 0.895, 95% CI = 0.852–0.941), and high social support (OR = 0.842, 95% CI = 0.772–0.919) were significant (Table 3).

Interaction of fall history with individual and environment characteristics on FOF by a hierarchical logistic regression model is shown in Table 4. A significant interaction was not observed between any individual or environmental variable on FOF of the participants with and without a fall history (Table 4).

## 4. Discussion

In this study, we compared the differences in predictors of FOF among older adults with or without a fall history from an ecologic aspect and examined whether the results were moderated by fall history in Korean older adults. We found that common predicting factors in both those with and without fall history in this study as follows; sex, and discomfort in the neighborhood environment were associated with FOF. However, the effects of interaction terms between fall history and the individual and environmental variables on FOF were not significant.

First, if considering the common individual factors for FOF in both those with and without a history of falling, previous studies [12,13,15] consistently report sex as predicting factors of FOF. Therefore, regardless of fall experience, it is important to manage FOF in older women. Second, when considering the common environmental factors for FOF in those with and without a history of falling, the older adults who had a higher level of discomfort with the neighborhood environment also had a higher level of FOF. This result is in close agreement with those of previous studies. Modification of home environment such as adequate lighting and handrails on the stairs and in the bathroom is one of the multifactorial recommendations to prevent falls [23]. Most old adults with FOF reported not going out alone and avoiding going outside because of FOF, and also had FOF both inside and outside of their homes [8]. FOF threatens autonomy, causes difficulty in walking [10], and makes older adults reluctant to leave their homes [8]. If a safe environment is not established both inside and outside of the house, activities may be hindered due to FOF in older adults [20,21,33]. Based on previous research, older adults reported that physical activity is most frequently performed in their homes [20], however, the results of a longitudinal study from 2008 to 2013 showed that frequency of outdoor falls was higher than that of indoor falls, and outdoor fall accidents gradually increased from 61.88% to 76.84% [27]. While having safe bike lanes [35,36] and traffic safety [36] promoted physical activity in the elderly [36], cracked streets and sidewalks, rainwater puddles, and bicycles in close proximity to pedestrians all contribute to fall risk and FOF [25]. Those kinds of environments are also barriers for physical activity in older adults [25]. Accordingly, it is necessary to establish a safe, friendly environment for older adults by evaluating the risk factors of falls and also by assessing the level of comfort when older adults are inside and outside their home and/or going to visit somewhere else.

Other than common factors of sex and discomfort with the neighborhood environment, age, education level, current driving status, subjective health status, trouble with vision, diabetes mellitus, arthritis, depression, accessibility to neighborhood facilities, and social support were related to FOF only for those without a fall history. The results of this study suggest that factors related to FOF between the group with fall experience and those without fall experience have not only common factors but also different factors. Therefore, to provide intervention for FOF, it is necessary to consider the common and differences of the factors of FOF of the group with fall experience and those without fall experience.

In our analysis, we verified that more individual and environmental factors were found to be associated with FOF among those without fall history compared to those with a fall history. Two factors for those with fall history compared to 12 factors for those without fall history were associated with FOF. This result is meaningful because there is no previous study to identify the factors related to FOF in older adults with and without fall history from an ecological perspective; however, further studies should consider other factors that were not included in this study for FOF in older adults with and without fall history. The findings from this study indicate that policy consideration is needed for environmental as well as individual factors of FOF among older adults with and without fall history in developing effective strategies to prevent FOF. Effective strategies to prevent FOF should consider both individual and environmental characteristics. Although individual factors such as sex cannot be modified, the environmental factors could be modified by promoting and/or changing the environment (e.g., improving accessibility to neighborhood environments and the social environment). To accomplish effective strategies, individual, community, and national cooperation are needed.

Among the individual factors related to FOF for only older adults without a fall history, it was suggested from previous research that age [12,13,15,27], educational level [15], subjective health status [13,15], trouble with vision [26], and depression [12,15] are consistently related to FOF. We speculate that the FOF in non-current driving older adults is higher than that of self-drivers because non-self-drivers tend to walk more and are therefore exposed to more environmental factors that could cause falls. Diabetes and arthritis are common chronic diseases in older adults, and FOF is common among people with diabetes [9] and/or musculoskeletal disease such as arthritis [10]. In addition, people with chronic disease-induced FOF could avoid or restrict activities [9,10]. Therefore, it is especially important to manage FOF and decrease falls for older adults with diabetes and arthritis.

Regarding environmental factors, the factors related FOF only those without a fall history were the physical environment such as accessibility to the neighborhood environment and the social environment such as social support. Previous studies have shown that better accessibility to shops [35,36], exercise facilities [36], and other facilities leads to older adults being engaged in more physical activities. It is inferred that older people have more difficulty walking due to normal aging, so a closer proximity of facilities such as the senior center and the gymnasium to homes of the elderly leads to a greater opportunity for them to participate in exercise and various programs. This in turn may have a positive effect on their physical activity and general health. Our results are consistent with previous studies in that good accessibility to the neighborhood environment such as the market, supermarket, hospital, government office, elderly welfare center, bus stop, and subway station was a protective factor against FOF. Therefore, it is important to ensure accessibility to the neighborhood environment for older adults.

The result of the current study is also consistent with those of earlier studies on social support, demonstrating that more social support leads to a lesser FOF, including having frequent encounters with close friends [12] and participating in frequent social activities, including sports, religious, and culture groups [12,26]; these social interactions also increase physical activity [36]. In addition, the WHO suggests that older adults tend to benefit from social programs and social support [19], mentioning that lack of social interaction increases risk for falling.

A study using nationally representative data of people age 65 years and above from the United States showed that social support and a more accessible physical environment were associated with fewer falls [22], similar to this study. In another study of community-dwelling older adults, FOF predicted lower quality of life [37] and was associated with anxiety and depression [38]. Social support provides a sense of trust and belonging, and the helpfulness and friendliness of family members and friends has a positive influence on the elderly [22].

Significant interactions with fall history and individual and environment factors of FOF were not observed in this study. We speculated that there are several reasons for this finding. First, in this study, the definition of fall included stumbling, slipping, and falling down, and the degree of fall was divided into two categories by dichotomizing yes or no. When the older adults responded to the question regarding falls, they might not clearly understand the meaning of fall by only considering fall as a hard fall onto the ground. Based on a review paper about the definition and measurement of fall, there are various definitions of fall, and no gold standard in fall measurement [39]. Therefore, an accurate definition and degree of FOF may play an important role in understanding the relationship between fall and FOF.

Second, FOF was initially described as “ptophobia—a phobic fear of falling,” which is a phobia in walking or standing after falls [40], and it was classified as post-fall syndrome [41]. FOF induces psychological trauma and negative effects on activity in older adults [42]. FOF is the result of falls [40,41], but it can also cause falls [2]. According to a recent 10-year longitudinal study, more than three times the number of falls in the last 12 months was a risk factor for FOF [12]. The results of this study should be interpreted with caution because this study design is cross-sectional. Therefore, a longitudinal study is needed to determine how fall history affects other related factors

Third, the measurement all environmental factors were based on self-report. Because there is often a discrepancy between subjective and objective elements of the environment [43], both measures are important to fully understand the relationships between the environment and FOF. Nevertheless, understanding the subjective environment is particularly important because FOF tends to be a psychological concern [37].

Fourth, we used a single question for measuring FOF because this was a secondary analysis of widely available data. Although a single question is commonly used [8,12,13] because it is simple and quick to use [44], it can have a relatively low sensitivity. In addition, it is difficult to confirm the accuracy of the response for the history of falling because the fall experience of the past one year was responded to the memory of the old adults. In future studies it would be desirable to use a reliable and valid instrument such as the Activities-specific Balance Confidence (ABC) Scale and/or the Falls Efficacy Scale (FES) to confirm our findings.

Despite these limitations, there are several strengths of this study. First, this is the first study that examined the correlation factors of FOF in older adults with fall history and without fall history using ecological models including environmental and individual factors. Second, this is the first study that examined the interactions between fall history and individual environmental factors on FOF in order adults. Finally, the results from this study came from a large sample based on nationally representative data.

## 5. Conclusions

In this study, we analyzed national survey data and confirmed individual and environmental factors of FOF between those with and without a fall history from an ecological aspect and examined whether individual and environmental factors differently affect the FOF according to fall history. There were both common and different correlations in individual and environmental factors and FOF between older adults with fall history and those without fall history. FOF showed different correlations with individual and environmental factors depending on the fall history, but fall history was not a potential moderator with individual and environmental characteristics on FOF. In future research, it will be necessary to standardize the definition, frequency, and degree of FOF and to investigate the interaction effects of fall history and ecological variables (individual and environmental) on FOF using a longitudinal study. An ecological model including individual and environmental factors should be considered when designing programs, conducting research, and forming decision policies related to FOF for older adults with and without a history of falling.

## Figures and Tables

**Table 1:** Differences in FOF according to the individual characteristics of the participants with and without a fall history (N = 7730).

Individual Variables		Classification	Total	With Fall History	Without Fall History	*t or χ*^2^
				(*n* = 1804)	(*n* = 5926)	
				*n* (%) or	*n* (%) or	
				M ± SD	M ± SD	
Demographic characteristics	Age (years)	65–74	4616 (59.7)	961 (53.3)	3655 (61.7)	40.631 ***
		≥75	3114 (40.3)	843 (46.7)	2271 (38.3)	
	Sex	Male	3154 (40.8)	503 (27.9)	2651 (44.7)	162.616 ***
		Female	4576 (59.2)	1301 (72.4)	3275 (55.3)	
	Marital status	Living with spouse	4851 (62.8)	943 (52.3)	3908 (65.9)	110.635 ***
		Living without spouse	2879 (37.2)	861 (47.7)	2018 (34.1)	
	Educational level (years)	0–6	5089 (65.8)	1356 (75.2)	3733 (63.0)	91.107 ***
		≥7	2641 (34.2)	448 (24.8)	2193 (37.0)	
	Public assistance	Yes	520 (6.7)	154 (8.5)	366 (6.2)	12.280 ***
		No	7210 (93.3)	1650 (91.5)	5560 (93.8)	
	Current driving status	Yes	1306 (16.9)	158 (8.8)	1148 (19.4)	110.964 ***
		No	6424 (83.1)	1646 (91.2)	4778 (80.6)	
Health-related characteristics	Subjective health status	Poor	5140 (66.5)	1469 (81.4)	3671 (61.9)	235.623 ***
		Good	2590 (33.5)	335 (18.6)	2255 (38.1)	
	Limitation of ADLs	Yes	291 (3.8)	126 (7.0)	165 (2.8)	67.344 ***
		No	7439 (96.2)	1678 (93.0)	5761 (97.2)	
	Limitation of IADLs	Yes	988 (12.8)	364 (20.2)	624 (10.5)	115.468 ***
		No	6742 (87.2)	1440 (79.8)	5302 (89.5)	
	Trouble with vision	Yes	3062 (39.6)	861 (46.7)	2201 (37.1)	64.788 ***
		No	4668 (60.4)	943 (53.3)	3725 (62.9)	
	Trouble with hearing	Yes	1676 (21.7)	494 (27.4)	1182 (19.9)	45.053 ***
		No	6054 (78.3)	1310 (72.6)	4744 (80.1)	
	Hypertension	Yes	4415 (57.1)	1103 (61.1)	3312 (55.9)	15.578 ***
		No	3315 (42.9)	701 (38.9)	2614 (44.1)	
	Diabetes mellitus	Yes	1682 (21.8)	441 (24.4)	1241 (20.9)	9.974 **
		No	6048 (78.2)	1363 (75.6)	4685 (79.1)	
	Arthritis	Yes	2728 (35.3)	856 (47.5)	1872 (31.6)	152.343 ***
		No	5002 (64.7)	948 (52.5)	4054 (68.4)	
	Depression ^†^			6.61 ± 4.57	4.57 ± 4.25	17.578 ***

** *p* < 0.01, *** *p* < 0.001. ^†^ The higher scores indicating the greater severity of depression. Note: The total number of participants is slightly different for the non-response items.

**Table 2:** Differences in FOF according to environmental characteristics of the participants with and without a fall history (N = 7730).

Environmental Variables	Classification	Total (N = 7730)	With Fall History	Without Fall History	
			(*n* = 1804)	(*n* = 5926)	*t or χ*^2^
			*n* (%) or M ± SD	*n* (%) or M ± SD	
Residential area	Urban	5296 (68.4)	1203 (66.7)	4093 (77.3)	3.642
	Rural	2434 (31.6)	601 (33.3)	1833 (22.7)	
Housing type	Apartment	2249 (29.1)	522 (28.9)	1727 (29.1)	0.029
	House	5481 (70.9)	1282 (71.1)	4199 (70.9)	
Discomfort with the neighborhood environment ^†^			2.13 ± 0.87	1.96 ± 0.96	6.802 ***
Accessibility to neighborhood facilities ^†^			2.28 ± 1.53	2.35 ± 1.48	−1.687
Social support ^†^			2.10 ± 0.86	2.19 ± 0.84	−4.040 ***

*** *p* < 0.001. ^†^ The higher scores indicate greater levels of the variable characteristics.

**Table 3:** Differences in FOF according to individual and environmental characteristics of the participants with and without a fall history (N = 7730).

Level	Variables	With Fall History	Without Fall History
	(Comparison Group)	OR (95% CI)	OR (95% CI)
Individual	Age (≥75 years)	1.680 (0.886–3.186)	2.047 (1.738–2.412) ***
	Sex (Female)	5.100 (2.488–10.456) ***	3.337 (2.810–3.962) ***
	Marital status	0.538 (0.259–1.119)	1.075 (0.898–1.288)
	Educational level (0–6 years)	1.158 (0.612–2.194)	1.221 (1.045–1.426) *
	Public assistance (Yes)	2.124 (0.456–9.893)	0.930 (0.648–1.336)
	Current driving status (Non-driver)	1.557 (0.773–3.139)	1.490 (1.250–1.775) ***
	Subjective health status (Poor)	1.910 (1.000–3.646)	1.651 (1.418–1.921) ***
	Limitation of ADLs (Yes)	0.554 (0.098–3.122)	1.029 (0.495–2.140)
	Limitation of IADLs (Yes)	1.145 (0.299–4.380)	1.347 (0.915–1.983)
	Trouble with vision (Yes)	1.923 (0.971–3.809)	1.409 (1.206–1.646) ***
	Trouble with hearing (Yes)	1.433 (0.675–3.044)	1.158 (0.953–1.406)
	Hypertension (Yes)	1.292 (0.718–2.325)	1.073 (0.931–1.236)
	Diabetes mellitus (Yes)	1.075 (0.521–2.218)	1.295 (1.074–1.562) **
	Arthritis (Yes)	1.772 (0.855–3.672)	1.490 (1.240–1.790) ***
	Depression ^†^	1.081 (0.995–1.174)	1.094 (1.070–1.118) ***
Environmental	Residential area (Rural)	1.230 (0.624–2.426)	0.968 (0.819–1.143)
	Housing type (House)	1.173 (0.632–2.175)	1.146 (0.979–1.341)
	Discomfort with neighborhood environment ^†^	1.387 (1.026–1.875) *	1.305 (1.211–1.406) ***
	Accessibility to neighborhood facilities ^†^	0.839 (0.687–1.025)	0.895 (0.852–0.941) ***
	Social support ^†^	0.995 (0.700–1.415)	0.842 (0.772–0.919) ***

* *p* < 0.05, ** *p* < 0.01, *** *p* < 0.001. ^†^ The higher scores indicate greater levels of the variable characteristics.

**Table 4:** Interaction of fall history with individual and environment characteristics on FOF by Hierarchical Logistic Regression.

Level	Variables (Comparison Group)	Model I Adjusted OR (95% CI)	Model II Adjusted OR (95% CI)
Individual	Age × Fall history	0.753 (0.398–1.426)	0.742 (0.389–1.417)
	Sex × Fall history	1.205 (0.627–2.318)	1.153 (0.595–2.233)
	Educational level × Fall history	1.011 (0.550–1.856)	0.952 (0.508–1.784)
	Current driving status × Fall history	1.058 (0.536–2.089)	1.014 (0.500–2.057)
	Subjective health status × Fall history	1.138 (0.599–2.160)	1.181 (0.618–2.255)
	Trouble with vision × Fall history	1.339 (0.684–2.623)	1.351 (0.686–2.663)
	Diabetes mellitus × Fall history	0.888 (0.427–1.847)	0.816 (0.391–1.701)
	Arthritis × Fall history	1.192 (0.570–2.492)	1.160 (0.550–2.445)
	Depression × Fall history	0.980 (0.905–1.061)	0.987 (0.909–1.072)
Environmental	Discomfort with the neighborhood environment × Fall history		1.051 (0.776–1.425)
	Accessibility to neighborhood facilities × Fall history		0.933 (0.768–1.133)
	Social support × Fall history		1.191 (0.833–1.704)

Model I was adjusted for socio-demographic (age, sex, educational level, current driving status) and health-related characteristics (subjective health status, trouble with vision, diabetes mellitus, arthritis, depression). Model II was adjusted for socio-demographic (age, sex, educational level, current driving status) and health-related (subjective health status, trouble with vision, diabetes mellitus, arthritis, depression) and environmental factors (discomfort with the neighborhood environment, accessibility to neighborhood facilities, social support).
